# Cognitively Stimulating Activities: Effects on Cognition across Four Studies with up to 21 Years of Longitudinal Data

**DOI:** 10.1155/2012/461592

**Published:** 2012-09-13

**Authors:** Meghan B. Mitchell, Cynthia R. Cimino, Andreana Benitez, Cassandra L. Brown, Laura E. Gibbons, Robert F. Kennison, Steven D. Shirk, Alireza Atri, Annie Robitaille, Stuart W. S. MacDonald, Magnus Lindwall, Elizabeth M. Zelinski, Sherry L. Willis, K. Warner Schaie, Boo Johansson, Roger A. Dixon, Dan M. Mungas, Scott M. Hofer, Andrea M. Piccinin

**Affiliations:** ^1^Edith Nourse Rogers Memorial Veterans Hospital, Geriatric Research, Education and Clinical Center, Massachusetts General Hospital, Harvard Medical School, 200 Springs Road, Bedford, MA 01730, USA; ^2^Departments of Psychology and Department of Neurology, University of South Florida, 4202 E. Fowler Avenue, Tampa, FL 33620, USA; ^3^Center for Biomedical Imaging, Medical University of South Carolina, 68 President Street, MSC 120, Charleston, SC 29425, USA; ^4^Department of Psychology, University of Victoria, P.O. Box 3050 STN CSC, Victoria, BC, Canada V8W 3P5; ^5^Division of General Internal Medicine, University of Washington, P.O. Box 359780, Harborview Medical Center, 325 Ninth Avenue Seattle, WA 98104, USA; ^6^Department of Psychology, California State University-Los Angeles 5151 State University Drive, Los Angeles, CA 90032, USA; ^7^Department of Food and Nutrition, and Sport Science, Department of Psychology, University of Gothenburg, P.O. Box 100, SE-405 30 Gothenburg, Sweden; ^8^Department of Psychology, University of Gothenburg, Box 500, SE 405 30, Gothenburg, Sweden; ^9^Ethel Percy Andrus Gerontology Center, Leonard Davis School of Gerontology, University of Southern California, Los Angeles, CA 90089-0191, USA; ^10^University of Washington, 180 Nickerson, Suite 206, Seattle, WA 98109, USA; ^11^Department of Psychology, University of Alberta, P217 Biological Sciences Building, Edmonton, AB, Canada T6G 2E9; ^12^Lawrence J. Ellison Ambulatory Care Center, University of California, Davis, 4860 Y Street, Ste 0100, Sacramento, CA 95817, USA

## Abstract

Engagement in cognitively stimulating activities has been considered to maintain or strengthen cognitive skills, thereby minimizing age-related cognitive decline. While the idea that there may be a modifiable behavior that could lower risk for cognitive decline is appealing and potentially empowering for older adults, research findings have not consistently supported the beneficial effects of engaging in cognitively stimulating tasks. Using observational studies of naturalistic cognitive activities, we report a series of mixed effects models that include baseline and change in cognitive activity predicting cognitive outcomes over up to 21 years in four longitudinal studies of aging. Consistent evidence was found for cross-sectional relationships between level of cognitive activity and cognitive test performance. Baseline activity at an earlier age did not, however, predict rate of decline later in life, thus not supporting the concept that engaging in cognitive activity at an earlier point in time increases one's ability to mitigate future age-related cognitive decline. In contrast, change in activity was associated with relative change in cognitive performance. Results therefore suggest that change in cognitive activity from one's previous level has at least a transitory association with cognitive performance measured at the same point in time.

## 1. Introduction

 With the rising proportion of older adults and increases in life expectancy [1], there has been increased interest in maintaining and promoting cognitive health in later life. Although declines in some domains of cognition are part of the natural course of aging [2, 3], sufficient evidence from prospective and observational studies indicates that the trajectories and outcomes of cognitive decline may be mitigated by participating in cognitively stimulating activities [4, 5]. Recent reviews of cognitive interventions suggest some potential benefits that may improve functioning in healthy older adults or slow decline in individuals with mild cognitive impairment (MCI) and those already affected with dementia [6–9]. Results from a meta-analysis of randomized controlled trials in healthy aging revealed a strong positive effect on cognition at immediate, medium-, and long-term followup after cognitive training [10]. Compelling results from large longitudinal studies have also shown that engagement in everyday cognitive activities predicts preserved cognition [11, 12] and decreases in incident Alzheimer's disease [13]. It is therefore not surprising that the market for “brain fitness” technologies, currently valued at $300 million, is projected to swell exponentially within this decade [14].

 A facet of this research that is relatively understudied involves examining the degree to which discrete types of everyday cognitive activity relate to change in specific cognitive domains over time. Most of the aforementioned trials incorporated training in multiple cognitive abilities and accordingly found support for cognitive training in general, but some reviews report less promising results for domain-specific training. Memory is frequently the targeted cognitive domain in many interventions, with training involving efforts to improve recall of newly learned information, including skills training, imagery, and mnemonic strategy use; however, meta-analyses reveal minimal efficacy for memory-focused techniques [15]. Investigations of the effectiveness of training in other cognitive abilities such as executive functions [16] and working memory [17] suggest that these have farther-reaching effects on cognitive function, but results are regarded as preliminary. Formal interventions employing cognitive skills training are rarely conducted outside of clinical trials, leaving observational studies as a valuable resource for evaluating potential benefits of everyday cognitive activities. Observational studies typically include self-report inventories of activities commonly regarded as cognitively stimulating, such as solving puzzles, listening to the radio, or reading books. While such studies are not experimental by design, they contribute a significant addition to the literature by assessing changes in accessible, everyday cognitive activities as these relate to change in cognitive abilities in a naturalistic setting. 

 Despite the promising body of work that has accumulated in recent years, definitive conclusions regarding the benefits of cognitive activity are precluded by several methodological concerns [11, 18]. Limitations include the inadequacy of activity assessments, psychometric variability of cognitive outcome measures, conceptual differences in the expected relationships between activities and specific cognitive domains, and insufficient assessment of moderating variables such as level of education and sex. While a study in which all these limitations are fully addressed has yet to be conducted, existing data from several longitudinal studies can be leveraged to disentangle some of these effects. In this paper, we intend to demonstrate that a coordinated analysis of four large longitudinal studies of aging can elucidate the benefits of changes in cognitive activity over time to performance trajectories in specific cognitive domains.

Thus, the purpose of this study was to examine the effects of self-reported everyday cognitive activities and changes in these activities on changes in four domains of cognition (reasoning, fluency, memory, and semantic knowledge) in longitudinal models that incorporate data from the Origins of Variance in the Oldest-Old: Octogenarian Twins Study (Octo-Twin), the Long Beach Longitudinal Study (LBLS), the Seattle Longitudinal Study (SLS), and the Victoria Longitudinal Study (VLS). This investigation was part of a larger coordinated effort to examine the effects of lifestyle activities on cognitive function across multiple large-scale longitudinal studies of aging that formed the basis of a meeting of the Advanced Psychometrics Methods in Cognitive Aging Workshop. The aim of this workshop was to use a common analytic protocol across studies from the Integrative Analysis of Longitudinal Studies on Aging (IALSA [19]) network. 

These studies were specifically selected based on their collection of cognitive, physical, and social activity data along with a range of cognitive functioning measures over multiple occasions. While the cognitive activity and cognitive function variables are not always identical, the subsets of variables in each study were chosen based on the rationale that they tapped similar domains at the construct level; specifically, we chose measures thought to tap fluid reasoning (Gf, i.e., verbal reasoning, block design, and verbal fluency measures), short-term memory (Gsm, i.e., immediate recall of a verbally presented story or word list), and crystallized knowledge (Gc, i.e., measures of vocabulary and acquired knowledge [20]). In some cases the measures are the same, but more often they differ, precluding statistical combination of indicators and outcomes between studies. However, we argue that concurrently analyzing the data with the same method provides opportunities for both strict and conceptual replication within the same study—an approach that to our knowledge has not been attempted previously. Thus, our goal was to build and implement a common model to each dataset to enable comparisons across all outcomes for the four longitudinal studies. The two primary hypotheses we tested were whether (1) cognitive activity at baseline would predict the trajectory of cognitive function over time and (2) change in cognitive activity would predict change in cognitive function over time.

## 2. Method

### 2.1. Multistudy Analysis Overview

We report a series of mixed effects models that included baseline and change in cognitive activity predicting cognitive function over up to 21 years of time in four large-scale longitudinal studies of older adults. Three of the four studies included in this analysis (LBLS, SLS, and VLS) specifically aimed to study healthy aging and only recruited community-dwelling older adults who were presumed to be cognitively normal at baseline. The fourth study, Octo-Twin, also included a largely cognitively normal sample of older adults, but those who did have dementia diagnoses at baseline (*n* = 98) were excluded from the present analysis. In order to model roughly equivalent cognitive outcomes across the four study samples in this coordinated effort, analyses included selected measures of reasoning, fluency, episodic memory, and semantic knowledge from the larger battery of tests included within each longitudinal study sample. In the Octo-Twin study, there was no fluency measure available, and we thus present only three of the four cognitive outcomes. These cognitive tasks were selected to represent a range of cognitive abilities from basic to more complex functions. Study sample characteristics and demographic, cognitive function, and cognitive activity measures are described below (Tables 1–4).

### 2.2. Origins of Variance in the Oldest-Old (Octo-Twin) Sample (Sweden) 

 The Octo-Twin study is based on the oldest cohort of the Swedish Twin Registry and includes 702 participants aged 80 years and older at the time of the first examination. All individuals with a dementia diagnosis at baseline were excluded from the analyses (*n* = 98). The total sample included 604 participants, of whom 572 had cognitive measures. The longitudinal design for survivors included a maximum of five measurement times at two-year intervals beginning in 1991–1993. The average rate of attrition from one test interval to the next was 20% (10% per year), primarily due to death.  Table 1 provides a summary of participant characteristics for the Octo-Twin participants included in this study. 

### 2.3. Octo-Twin Materials and Procedure 

#### 2.3.1. Octo-Twin Cognitive Ability Measures

*Reasoning* was assessed using block design [21], in which participants are presented with red and white blocks and instructed to assemble the blocks to reproduce a design portrayed on a card within a predetermined time limit. As previously mentioned, the Octo-Twin study did not have a measure of fluency so this cognitive domain was not analyzed and compared with fluency results from the three other longitudinal studies. *Memory *was assessed using the Prose Recall test in which participants were asked for immediate free recall of a brief (100 word) story that had a humorous point [22]. Responses were coded for the amount of information recalled in a manner similar to the scoring of story units in the Wechsler Memory Scale Logical Memory test [23]. *Semantic knowledge *was assessed using the Swedish version of the WAIS Information Task [24], which requires participants to provide answers to questions assessing acquired knowledge of facts [25].

#### 2.3.2. Octo-Twin Cognitive Activity Measure

 The cognitive activity measure was based on self-report of engagement in six cognitively stimulating activities including playing games (e.g., chess and bridge), completing crossword puzzles, reading literature, writing, conducting genealogical research, or any otherdocumentation,studies, or other mentally demanding activity (e.g., handicraft), each rated dichotomously as “no” (0) or “yes” (1). Participants were also asked if they “train their memory or keep their mind active” rated as “no” (0), “yes, to a certain degree” (1), or “yes, definitely” (2). A composite score for cognitive activity was created by summing responses across items (range = 0–8). Change in cognitive activity was computed by subtracting the cognitive activity score at baseline from all subsequent activity scores.

### 2.4. Long Beach Longitudinal Study Sample (California, USA)

The LBLS was started in 1978 in Long Beach, California, with participants recruited from the Family Health Plan Health Maintenance Organization (HMO) who were primarily from Long Beach and Orange Counties. Panel 1 included 583 individuals aged 28–36 or 55–87. The ethnic composition of the older group (98% Caucasian) was similar to the 65+ population for the area based on the 1970 census. Panel 2, initiated in 1992, included 633 contacted from the same HMO (64 were excluded due to frank dementia, serious sensory, or neurological problems). In order to include the same measures as those in the Seattle Longitudinal Study, LBLS Panel 1 (*n* = 106) and Panel 2 (*n* = 631) data from 1994 to 2003 were used in the current analysis. During this period, data were collected at 3-year intervals. Only visits occurring at age of 55 or older were included in this study (baseline *n* = 561). Dementia incidence is not known.

Demographic information and descriptive statistics for the sample are presented in Table 2. The table displays the number of participants at each test occasion that completed the four cognitive measures and the retention rates for those measures from one testing to the next (top line). Collapsed across measures and testing, the average retention rate from one testing to the next was 55.8% or 17% per year. Age and education increased from the first to the fourth test occasions, suggesting that the sample became more selective over time. Similar patterns of selection were observed for the cognitive measures of reasoning, memory, fluency, and semantic knowledge. 

### 2.5. LBLS Materials and Procedure

#### 2.5.1. LBLS Cognitive Ability Measures

*Reasoning* was indexed as a composite score of the Schaie-Thurstone Adult Mental Abilities Test (STAMAT [26]) Letter and Word Series tests. In Letter Series, participants viewed a series of letters (e.g., a b c c b a d e f f) and were asked to discover the rule that governs the series by identifying the letter from an array of four possible responses that should come next in the series. Participants were to complete as many of the 30 items as possible within six minutes. Word Series was a parallel test to Letter Series but the letters were replaced with months (e.g., January) and days of the week (e.g., Monday). *Fluency *was measured using Word Fluency, in which participants were instructed to write down as many words as possible in five minutes that begin with a specified letter “s.” Participants were instructed that they could not use proper nouns or create words by changing endings of other listed words (e.g., if the letter was “w” and you already said “want,” you should not also say “wants,” “wanting,” or “wanted”). *Memory* was measured using immediate written recall of a list of 20 concrete high-frequency nouns studied for 3.5 minutes. *Semantic knowledge* was assessed using the STAMAT Recognition Vocabulary test. Participants were given a word and asked to circle a synonym of that word from four possible alternatives. The test included 50 items completed within a 5 minute time limit.

#### 2.5.2. LBLS Cognitive Activity Measure

The cognitive activity measure was derived from a modified version of the Life Complexity Scale (LCS), originally developed for the Seattle Longitudinal Study [27]. The modified scale consisted of six items from the LCS: educational activities, leisure reading, playing musical instruments, writing letters, playing games, and cultural activities. Participants were asked to record the number of “hours per week on average” they spent doing each activity. Due to extreme variability in reported hours observed within and between items, responses were dichotomized for the present analysis with those who reported no time spent on a given activity coded as 0 and those who reported one or more hours of activity coded as 1. Items were summed to create a composite measure of cognitive activity (range = 0–6). Change in cognitive activity was computed by subtracting the cognitive activity score at baseline from all subsequent activity scores.

### 2.6. Seattle Longitudinal Study Sample (Washington, USA)

The SLS was initiated in 1956 in Seattle, Washington, and includes eight samples recruited from a local HMO at seven-year intervals and followed longitudinally every seven years (total *n* across all study samples = 4,854). The current analysis includes data from participants in the study from 1984 to 2005 (total *n* across 1984–2005 study samples = 2,040) and includes longitudinal data for up to four testing occasions. This subset of the larger study was selected due to changes in measures used over the course of the entire study in order to have equivalent measures of cognition and activity at each time point and with the LBLS. Only visits occurring at age 55 or older were included in our analyses, yielding a total of 1,649 participants at baseline. Baseline was defined as each participant's first study visit, and time was measured in all analyses as years in study (coded as 0, 7, 14, and 21). Attrition during these 7-year intervals was approximately 50%, or about 7% per year. Dementia prevalence and incidence are not known. See Table 3 for SLS participant characteristics over the four waves of data analyzed here. 

### 2.7. SLS Materials and Procedure

#### 2.7.1. SLS Cognitive Measures

*Reasoning* was assessed with the Word Series test from the Schaie-Thurstone Adult Mental Abilities Test (STAMAT [26]), in which participants were provided with a printed word series and instructed to choose the next word in the series in multiple-choice format by identifying the rule that governed a series. The test consisted of 30 items, and total score was based on number of correct responses completed in 6 minutes. *Fluency *was assessed with the Word Fluency test from the Primary Mental Abilities test [28], in which participants were asked to write down words beginning with the letter “s” following a rule set (do not use proper nouns and do not use different conjugations of the same word). Total score was based on number of correct responses generated in 5 minutes. *Memory* was assessed with a task in which participants were asked to study a list of 20 printed words for 3.5 minutes and provide immediate written recall of the items. *Semantic knowledge *was assessed with the Educational Testing Service (ETS) test of Advanced Vocabulary, in which participants were asked to identify synonyms for printed words from 5 choices [29]. Total score was based on number of correctly identified synonyms out of 36 test items completed within 4 minutes.

#### 2.7.2. SLS Cognitive Activity Measure

The cognitive activity measure was derived by summing dichotomized test responses to five cognitive activity items (reading, educational activities, music, writing, and cultural activities) from a modified version of the Life Complexity Scale [27]. Cognitive activity change was computed by subtracting baseline activity from each follow-up activity measure. 

### 2.8. Victoria Longitudinal Study Sample (British Columbia, Canada)

The VLS was begun in the 1986 in Victoria, British Columbia, and consists of three cohorts started in 1986, 1992, and 2001, respectively, followed longitudinally at 3-year intervals. Longitudinal data used in this study were from Samples 1 (baseline *n* = 484) and 2 (baseline *n* = 530). For this investigation, data from seven waves of Sample 1 and five waves of Sample 2 were included in analyses. Approximately 25% of the sample was lost to follow up at each wave or 8% per year. Dementia prevalence and incidence are not known. Relevant demographic information regarding the study sample is provided in Table 4. 

### 2.9. VLS Materials and Procedure

#### 2.9.1. VLS Cognitive Ability Measures

*Reasoning* was indexed by Letter Series [30] in which participants were presented with a series of letters and asked to identify the next letter in the sequence that was consistent with the sequence rule. *Fluency* was measured by performance on a similarities task [30]. In this timed task, participants were presented with target words and asked to write as many words as possible with the same or nearly the same meaning within 6 minutes. *Memory *was indexed using a 30-item noun list learning task comprised of five semantic categories. Participants studied the word list for 2 minutes followed by a 5-minute free recall task [30]. *Semantic knowledge* was assessed using a 54-item recognition vocabulary test. This task was adapted from the ETS Kit of Factor Referenced Tests [29]. 

#### 2.9.2. VLS Cognitive Activity Measure

The cognitive activity measure included a subset of items from the VLS Activity Lifestyle Questionnaire [3]. The 27 items comprising the Novel Information Processing scale were selected due to the cognitively stimulating nature of the activities. For each item, participants indicated the frequency of engagement in that activity over the past two years on a scale from 0 to 9 (i.e., *never, less than once a year, about once a year, 2 or 3 times a year, about once a month, 2 or 3 times a month, about once a week, 2 or 3 times a week, *and *daily)*. Individual item distributions were reviewed, and 11 of the 27 original items with little to no variability were eliminated. The remaining items were a priori hypothesized to fall into three general types of activities: those involving what we termed “Communication,” “Computations,” or “Conundrums.” Confirmatory factor analysis using Mplus version 6.0 [31] was conducted to test a three-factor model including six items indexing Communication (enrolling in college courses, giving a talk, attending lectures, studying a second language, writing, and writing letters specifically), five items indexing Computation (balancing a check book, performing mathematical calculations, working on taxes, engaging in business activity, and using a calculator), and five items indexing Conundrums (engaging in crosswords, chess/checkers, knowledge games, word games/scrabble, and jigsaw puzzles). Fit criteria were the comparative fit index (CFI) and the root mean squared error of approximation (RMSEA), where criteria for excellent fit include CFI > 0.95 and RMSEA < 0.05 [32]. Allowing for within-factor residual correlations, the model demonstrated acceptable fit (CFI = 0.95, RMSEA = 0.04). The factor scores generated by this analysis were then used as the primary predictor variables in three separate mixed effects models. 

### 2.10. General Analytic Approach

 The current analysis was conducted as part of a larger effort to examine the effects of lifestyle activities on cognitive function using the same analytic approach across studies from the Integrative Analysis of Longitudinal Studies on Aging (IALSA) network [19], and models were selected in part to maintain consistency across lifestyle activities. Across all four studies, we examined common demographic covariates including age (in years), years of formal education, and sex (coded as 0 = male, 1 = female). Age and education were mean centered to their respective study's baseline mean value. In order to maximize use of all available data, we defined baseline as the first study visit for each participant with available cognitive activity data. We analyzed the data with mixed effects modeling using Stata software, version 12 (StataCorp, 2011) and restricted maximum likelihood (REML) estimation, random slopes and intercepts, and an unstructured covariance matrix. In the Octo-Twin study, participants were nested within their twin pair. In the VLS, we controlled for enrolment cohort. Model assumptions were verified by examining residuals computed using predicted values that included the random effects. Separate models were fit for each of the four cognitive measures. We defined the criterion for significance as *P* < 0.05. While we recognize that this criterion may be viewed as liberal, given the large number of comparisons across all statistical models in our analysis, we assert that this approach is warranted in this study as we are representing results from four independent longitudinal studies following similar statistical procedures for each. Thus, the emphasis in this paper is replication of the pattern of results across the studies. In this way, the “strictness” of the evaluation of the effects comes from noting whether a particular effect is replicated across the different samples. The “familywise” alpha rate is that which occurs within each study, not across all of them together. 

## 3. Results

An initial 19-term model included the following terms: (1) baseline age, (2) sex, (3) education, (4) baseline activity, (5) baseline activity × age, (6) baseline activity × sex, (7) baseline activity × education, (8) individually defined time since baseline, (9) time × baseline age, (10) time × sex, (11) time × education, (12) time × baseline activity, (13) time × baseline activity × baseline age, (14) time × baseline activity × sex, (15) time × baseline activity × education, (16) change in activity from baseline (activity change), (17) activity change × baseline age, (18) activity change × sex, and (19) activity change × education. This full model was evaluated in each study data set independently, and terms that were not significant in any of the four studies were dropped in order to present a parsimonious set of results that retained the fullest set of parameters found in any study. This process eliminated 7 of the 19 terms, including all 3-way interactions, and four of the 2-way interactions, including the interactions between activity change and age, sex, or education, as well as the interaction between baseline activity level and sex. This resulted in a final model that included 12 terms summarized in Table 5. As a proper meta-analytic summary would require identical measures across a larger number of studies, we rely on straightforward comparison of the conclusions derived from each study.

### 3.1. Baseline Covariates and Cross-Sectional Relationships

There was a significant relationship between self-reported cognitive activity at baseline and baseline performance on tests of cognitive abilities across all measures and studies but the LBLS, which did not find this relationship in the reasoning and memory models. Overall, these findings suggest that participants who were more cognitively active at baseline tended to have better cognitive performance. One of the studies (VLS) included three distinct measures of cognitive activity—those involving Communication (e.g., writing), Computations (e.g., managing finances), and Conundrums (e.g., completing crossword puzzles)—enabling us to determine if specific cognitive activities were differentially related to the cognitive outcomes. While all three types of cognitive activities showed significant cross-sectional relationships with cognitive outcomes (all *P* < 0.001), the strongest relationships with cognitive function were found for Conundrums, followed by Computation and Communication. 

Older age was associated with lower baseline performance across all studies on measures of reasoning, fluency, and memory. In contrast, the relationship between age and baseline performance on semantic knowledge measures was inconsistent, with LBLS and Octo-Twin results suggesting lower performance in older age, SLS showing no age differences, and VLS suggesting that older age was associated with better performance. Baseline associations between sex and cognitive performance showed a consistent relationship across studies for all memory outcomes, with women consistently performing higher than similar aged men. SLS and LBLS women additionally performed higher than men on reasoning and fluency measures. Across other cognitive outcomes, baseline associations between performance and sex were less consistent, with VLS women performing better on fluency in the Computations model and Octo-Twin women performing lower than men on semantic knowledge. Higher education was consistently associated with higher baseline cognitive performance across all studies and cognitive outcomes. 

Two baseline covariate interaction terms were retained in the final model, and both showed inconsistent relationships across studies and outcome measures: the age by baseline cognitive activity interaction term was significant in the VLS memory models and the Conundrums/semantic knowledge model. There was a similarly significant interaction between baseline age and activity level in the LBLS reasoning model (*P* < 0.05). The education by baseline cognitive activity interaction term in the VLS Communication models for reasoning, fluency, and semantic knowledge was significant, suggesting that those with lower education had a higher association between baseline activity and cognitive test performance. The VLS Computation and Octo-Twin models for semantic knowledge also showed this relationship. 

### 3.2. Longitudinal Relationships

Across all studies and cognitive outcomes, there was, with one exception (VLS computation with reasoning), no evidence for baseline level of cognitive activity predicting change in cognitive outcomes over time. There was, however, a consistent positive relationship between change in cognitive activity from baseline and within person variability in cognitive outcomes across nearly all cognitive outcomes in all four studies. Specifically, after accounting for the expected linear within person trajectories, variation in cognitive activity was significantly related to variation in performance on all measures in all studies except reasoning and fluency in LBLS and reasoning and memory, in the case of Conundrums only, in VLS. 

Within-person declines were seen over time across all studies and all cognitive outcomes except LBLS fluency. Older participants declined faster compared to younger participants on all VLS, SLS, and LBLS cognitive outcome measures except LBLS memory. Evidence for differential decline in older participants was not seen in Octo-Twin, which has a much narrower age range. Women declined less than men on fluency measures in the SLS and VLS Computations models and on semantic knowledge measures in the SLS and Octo-Twin study. Level of education was not a significant predictor of rate of cognitive decline in all but one study (LBLS) and one outcome measure (reasoning, coefficient = −0.06, *P* < 0.01). 

## 4. Discussion

Our results provide compelling evidence across four longitudinal studies that changes in everyday cognitive activity level tracks with variation in multiple aspects of cognitive function. In three of the four studies (Octo-Twin, SLS, and VLS), participants reported engaging in fewer cognitive activities over time. In the fourth study (LBLS), participants endorsed a slight increase in average number of cognitive activities over time, which was likely due to differential retention of higher functioning individuals. While the overall trend was for participants to report slightly less cognitive activity at each follow-up visit in all but the LBLS sample, there was actually considerable variability in activity change scores, with some participants in each study reporting increased cognitive activity at follow-up visits relative to their baseline levels. 

These results suggest that there is an increased risk of cognitive decline for individuals whose engagement in cognitive activities decreases over time relative to their baseline levels, and, conversely, the results suggest that increases in cognitive activity from baseline are associated with better than expected cognitive performance. Cognitive activity change appeared to most consistently track with variation in semantic knowledge, as the activity change term was significant in all six models. Strong evidence of activity change tracking with fluctuations in memory and fluency was also indicated, as five of six models had significant activity change terms in the memory models and in four of the five fluency models. Activity change was significantly related to variation in reasoning in four of the six models, making the models with reasoning outcomes the least consistent relative to models with the other cognitive outcomes. That two of the four inconsistent findings occurred in LBLS, which had the most similarity with SLS, in terms of both measures and sampling, suggests that some other factor, such as attrition, may be responsible for these differences. It is interesting to note, however, that the standard deviation of reported activity level did not differ from that of SLS. The lack of association with reasoning and memory for one of the three VLS activity variables (Conundrums) could be due to chance, although this finding may also suggest that changes in level of engagement on tasks involving problem solving are less related to changes in reasoning and memory function than they are to changes in fluency and semantic knowledge.

Across studies, with the exception of VLS Computation with reasoning, there were no significant relationships between baseline cognitive activity and change in cognition over time, suggesting that level of cognitive activity at an earlier point in time is not related to subsequent cognitive decline. Thus, these results do not demonstrate that level of engagement in cognitively stimulating activities earlier in older adulthood can somehow increase one's cognitive reserve or ability to maintain cognitive function in spite of age-related brain changes [33]. Nonetheless, our results do have important clinical implications in that they suggest that individuals who exhibit changes from a previous level of cognitive activity can be expected to have associated fluctuations in cognitive performance, or vice versa.

In terms of cross-sectional relationships, all studies provide evidence for activity/cognition relationships, and the VLS results allow us to conclude that level of engagement in cognitive activities involving what we termed “Conundrums” (e.g., playing chess, completing crossword puzzles) are most strongly and consistently related to concurrent function across cognitive domains, but evidence for relationships between engagement in activities involving Computations (e.g., balancing a check book) and Communication (e.g., writing letters) was also demonstrated. Thus, while the data do not provide particularly compelling evidence that engagement in one type of cognitively stimulating activity is preferable, activities involving novel information processing appear to be most related to concurrent cognitive function, a finding that is consistent with the extant literature [34].

The lack of evidence for cognitive activity level at baseline predicting cognitive decline over time in some respects may be interpreted as discouraging, as it implies that older adults who more frequently engage in cognitive activities may not be influencing the trajectory of their cognitive function in the coming years. However, across all studies, change in level of cognitive activity from baseline generally followed a normal distribution, with considerable portions of each sample reporting an increase in level of cognitive activity from baseline levels. The positive association between cognitive activity change and the cognitive outcomes across studies thus suggests that individuals who increase their cognitive activities may be effectively reducing age-related cognitive decline. 

Our results demonstrate that older age is associated with faster decline, which supports the overall validity of our approach and suggests that we are detecting relevant change. The finding that education was not predictive of rate of cognitive decline with one exception (the LBLS reasoning model) suggests that education is not protective or predictive of a faster decline in normal aging. These multistudy results build upon findings from a recent paper using data from one of the studies (VLS) included in the current paper [35], in which the authors conclude that the relationship between education and cognitive performance is merely a cross-sectional relationship between level of education and cognitive function, and that longitudinal models that covary for baseline cognitive function are in effect creating a statistical artifact that is seen as an effect of education on rate of decline [34]. However, it is important to note that all studies included in the current analysis were designed to characterize normal cognitive aging, and results are not directly comparable to studies examining the effect of education level or cognitive reserve on the incidence and rate of decline in Alzheimer's disease [33].

The current study has many strengths, including the large sample sizes and multinational representation in our study samples, which improves the generalizability of the findings. In addition, the inclusion of four separate studies with unique sample characteristics, methodologies for recruitment, different methods for measuring cognitive activity and cognitive function, and differing frequency and length of followup, all serve to minimize the likelihood that these findings are spurious. When results across such a coordinated analysis are inconsistent, any one of these differences between studies could be responsible for discrepancies and reflect a limitation of the design. For example, the inconsistencies in the relationships between baseline covariates and their interactions (e.g., sex and age with baseline activity level) highlight a weakness of our study design. Inconsistencies could also be attributable to the heterogeneity in the activity measures used across the four longitudinal studies, as the scales included different items with different response ratings, yielding restricted ranges of responses on some measures. It is also possible that the inconsistencies are due to differences in the cognitive outcomes used in the different studies, or any number of other differences in the methodologies across studies. However, it is important to note that when the model results demonstrate consistent patterns across studies despite variations in methodology, the heterogeneity of measures and sampling methods becomes a major strength of the multi-study approach, as there is improvement in the reliability of conclusions that can be drawn from the results, relative to the typical single-study design. 

Perhaps the most obvious limitation inherent in the observational design of all studies included in this investigation is that conclusions implying causality cannot be inferred from these results. Specifically, while an increase in cognitive activity from baseline was associated with better than expected cognitive performance, and, conversely, activity decrease was associated with worse than expected performance, it is not possible to conclude that change in activity level was the cause for change in rate of cognitive decline. An alternative explanation is that decreases in level of cognitive activity from baseline levels observed in this study result from deteriorating cognitive functions rather than cause it. Put simply, this study design does not answer whether completing crossword puzzles reduces one's risk of cognitive decline or if cognitive decline reduces the likelihood that one will complete crossword puzzles. In addition, this study does not address the protective effects of cognitive activity for incident dementia or Alzheimer's disease. While there is a large body of the literature examining the beneficial effects of cognitive activity in reducing dementia risk (e.g., [36]), the studies included in the current investigation were based on normal cognitive aging, and individuals with dementia diagnoses were excluded from the present analysis.

What these results impart, however, is that regardless of the causal mechanisms underlying these changes, the associations between cognitive activity and cognitive outcomes in this study are in directions that are intuitively and scientifically consistent with prior literature. This fact, coupled with the large-scale naturalistic, observational design of this study, lends credence to the burgeoning literature that directly examines the causal effect of cognitive activity on cognitive outcomes. Extension of this work in populations at great risk for dementia, or with individuals already diagnosed with neurodegenerative diseases, remains a worthwhile goal.

## Figures and Tables

**Table 1:** Octo-Twin participant characteristics.

	Year of testing				
Measure	Baseline	Year 2	Year 4	Year 6	Year 8
	(*n* = 572)	(*n* = 470)	(*n* = 361)	(*n* = 274)	(*n* = 194)
Retention from previous testing (%)		82.2	76.8	75.9	70.8
Age [M (SD)]	83.3 (3.0)	85.2 (2.8)	86.9 (2.5)	88.8 (2.5)	90.7 (2.4)
Education [M (SD)]	7.2 (2.4)	7.3 (2.4)	7.3 (2.3)	7.2 (2.1)	7.2 (2.3)
Sex, female [*n* (%)]	369 (65)	304 (65)	235 (65)	195 (71)	144 (74)
Reasoning [M (SD)]	11.5 (7.1)	11.4 (7.2)	11.4 (7.1)	10.8 (7.2)	10.3 (7.3)
Memory [M (SD)]	9.6 (4.0)	9.4 (4.2)	9.2 (4.4)	9.2 (4.7)	9.0 (4.4)
Semantic knowledge [M (SD)]	28.1 (11.1)	28.6 (11.2)	27.5 (12.4)	26.7 (13.0)	26.2 (11.4)
Cognitive activity [M (SD)]	2.1 (1.7)	1.7 (1.6)	1.3 (1.4)	1.2 (1.4)	1.1 (1.3)
Activity change [M (SD)]	—	−0.3 (1.3)	−0.6 (1.3)	−0.8 (1.5)	−1.1 (1.6)

M: mean; SD: standard deviation. The theoretical ranges for each measure with a defined upper limit are as follows: reasoning = 0–42, Memory = 0–16, semantic knowledge = 0–44, and cognitive activity = 0–8.

**Table 2:** LBLS participant characteristics.

	Year of testing			
Measure	Baseline	Year 3	Year 6	Year 9
	(*n* = 561)	(*n* = 292)	(*n* = 144)	(*n* = 101)
Retention from previous testing (%)		52.0	49.3	70.1
Age [M (SD)]	73.7 (9.2)	75.4 (8.7)	75.1 (8.0)	76.1 (7.1)
Education [M (SD)]	13.7 (3.0)	13.9 (2.8)	14.2 (2.7)	14.2 (2.7)
Sex, female [*n* (%)]	285 (51)	146 (50)	70 (49)	51 (51)
Reasoning [M (SD)]	22.3 (11.7)	23.9 (11.5)	25.4 (11.6)	25.2 (11.1)
Fluency [M (SD)]	32.4 (11.6)	33.7 (11.1)	33.3 (13.3)	34.4 (11.7)
Memory [M (SD)]	11.4 (4.0)	11.6 (4.3)	11.6 (4.5)	11.2 (4.6)
Semantic knowledge [M (SD)]	38.5 (10.3)	39.5 (9.6)	40.7 (9.0)	39.7 (9.8)
Cognitive activity [M (SD)]	2.5 (1.3)	2.8 (1.4)	2.7 (1.3)	2.6 (1.3)
Activity change [M (SD)]	—	0.2 (1.3)	0.2 (1.4)	−0.2 (1.3)

M: mean; SD: standard deviation. The theoretical ranges for each measure with a defined upper limit are as follows: education = 0–20, reasoning = 0–30, memory = 0–20, semantic knowledge = 0–36, and cognitive activity = 0–6.

**Table 3:** SLS participant characteristics.

	Year of testing			
Measure	Baseline	Year 7	Year 14	Year 21
	(*n* = 1649)	(*n* = 939)	(*n* = 445)	(*n* = 178)
Retention from previous testing (%)		56.4	47.7	40.8
Age [M (SD)]	67.1 (8.2)	72.9 (7.3)	77.9 (6.4)	81.8 (4.9)
Education [M (SD)]	14.6 (2.9)	14.7 (2.8)	14.8 (2.7)	14.8 (2.8)
Sex, female [*n* (%)]	859 (52)	502 (54)	255 (57)	108 (60)
Reasoning [M (SD)]	15.6 (5.8)	15.2 (5.6)	14.3 (5.5)	14.0 (5.3)
Fluency [M (SD)]	38.6 (12.8)	37.5 (13.1)	36.7 (12.7)	38.8 (14.5)
Memory [M (SD)]	12.5 (4.0)	12.0 (4.1)	11.5 (4.2)	11.6 (4.0)
Semantic knowledge [M (SD)]	25.0 (6.7)	25.3 (6.6)	25.8 (6.2)	25.8 (5.9)
Cognitive activity [M (SD)]	2.4 (1.2)	2.5 (1.2)	2.5 (1.2)	2.3 (1.2)
Activity change [M (SD)]	—	−0.1 (1.1)	−0.2 (1.1)	−0.5 (1.3)

M: mean; SD: standard deviation. The theoretical ranges for each measure with a defined upper limit are as follows: education = 0–20, reasoning = 0–30, memory = 0–20, semantic knowledge = 0–36, and cognitive activity = 0–5.

**Table 4:** VLS participant characteristics.

	Year of testing						
Measure	Baseline	Year 3	Year 6	Year 9	Year 12	Year 15	Year 18
	(*n* = 1011)	(*n* = 733)	(*n* = 579)	(*n* = 417)	(*n* = 286)	(*n* = 91)	(*n* = 52)
Retention from previous testing (%)^a^	—	73	79	72	69	72	57
Age [M (SD)]	68.8 (6.8)	71.4 (6.7)	73.7 (6.5)	76.6 (6.0)	79.3 (5.2)	82.2 (4.6)	85.1 (3.6)
Years of education at baseline [M (SD)]	14.9 (3.3)	15.4 (3.2)	15.7 (3.1)	15.8 (3.1)	15.8 (3.1)	15.2 (3.1)	14.8 (2.8)
Sex, female [*n* (%)]	642 (63.5)	459 (62.6)	353 (61.0)	256 (61.4)	177 (61.9)	60 (65.9)	35 (67.3)
Reasoning [M (SD)]^b^	11.1 (4.6)	11.6 (4.2)	10.3 (4.7)	10.3 (4.6)	9.9 (4.6)	7.5 (4.7)	6.5 (4.2)
Fluency [M (SD)]	17.5 (4.4)	17.9 (4.4)	17.7 (4.4)	17.2 (4.8)	16.3 (4.9)	14.7 (5.8)	13.8 (5.4)
Memory [M (SD)]^c^	13.7 (5.9)	14.6 (6.0)	14.7 (6.1)	14.8 (6.4)	11.8 (5.5)	13.0 (6.3)	—
Semantic knowledge [M (SD)]	43.6 (7.5)	44.6 (6.3)	44.3 (6.0)	44.2 (5.9)	43.6 (5.7)	42.7 (7.1)	42.6 (6.6)
Cognitive activity [M (SD)]							
Communication	0.1 (0.8)	0.1 (0.8)	0.0 (0.8)	−0.1 (0.8)	−0.2 (0.8)	−0.4 (0.8)	−0.4 (0.7)
Computation	0.1 (0.8)	0.0 (0.8)	0.0 (0.8)	0.0 (0.8)	−0.1 (0.8)	−0.3 (0.7)	−0.5 (0.7)
Conundrums	0.0 (0.8)	0.0 (0.8)	0.0 (0.8)	0.0 (0.8)	0.0 (0.8)	−0.2 (0.9)	−0.2 (0.8)
Activity change [M (SD)]							
Communication	—	0.0 (0.5)	−0.2 (0.6)	−0.3 (0.6)	−0.4 (0.6)	−0.5 (0.6)	−0.6 (0.5)
Computation	—	−0.1 (0.6)	−0.2 (0.6)	−0.3 (0.6)	−0.4 (0.7)	−0.4 (0.7)	−0.5 (0.8)
Conundrums	—	0.0 (0.6)	−0.1 (0.6)	−0.1 (0.7)	−0.2 (0.6)	−0.3 (0.8)	−0.4 (0.7)

M: mean; SD: standard deviation. The theoretical ranges for each measure with a defined upper limit are as follows: reasoning 0–20, memory 0–30, and semantic knowledge 0–54. The cognitive activity scores are on a normal metric with means of approximately 0 and SD of approximately 0.8.

^a^The 1986 cohort was followed for up to 18 years and the 1993 cohort for up to 12.

^b^The reasoning measure was not given until year 6 for the 1986 cohort.

^c^The memory measure was not given in year 18.

**Table 5:** Mixed effects model summaries across four studies with baseline cognitive activity and activity change predicting four cognitive outcomes.

	Octo-Twin			LBLS			SLS			VLS								
Reasoning										Communication			Computation			Conundrums		
	*b*	SE	*P*	*b*	SE	*P*	*b*	SE	*P*	*b*	SE	*P*	*b*	SE	*P*	*b*	SE	*P*
Intercept	11.17	0.64	<0.001	20.98	0.58	<0.001	14.83	0.18	<0.001	9.60	0.58	<0.001	9.06	0.56	<0.001	9.64	0.57	<0.001
Age	−0.32	0.10	0.002	−0.60	0.05	<0.001	−0.35	0.01	<0.001	−0.25	0.02	<0.001	−0.22	0.02	<0.001	−0.24	0.02	<0.001
Sex	0.10	0.59	0.862	2.58	0.82	0.002	1.40	0.24	<0.001	−0.12	0.31	0.700	0.42	0.31	0.170	−0.19	−0.19	0.300
Education	0.39	0.14	0.015	1.24	0.15	<0.001	0.48	0.05	<0.001	0.29	0.05	<0.001	0.25	0.05	<0.001	0.32	0.05	<0.001
Activity	1.46	0.18	<0.001	0.02	0.33	0.956	0.34	0.11	0.001	0.45	0.20	0.024	1.24	0.19	<0.001	0.94	0.19	<0.001
Age × activity	0.05	0.06	0.389	0.09	0.04	0.020	0.01	0.01	0.286	0.00	0.02	0.876	0.04	0.02	0.078	0.02	0.02	0.447
Education × activity	−0.05	0.06	0.453	0.04	0.10	0.702	−0.05	0.03	0.168	−0.12	0.05	0.020	0.00	0.05	0.923	0.05	0.05	0.404
Time	−0.45	0.09	<0.001	−0.50	0.08	<0.001	−0.25	0.02	<0.001	−0.24	0.03	<0.001	−0.25	0.03	<0.001	−0.25	0.03	<0.001
Age × time	−0.02	0.02	0.274	−0.03	0.01	0.000	−0.01	0.00	<0.001	−0.01	0.00	<0.001	−0.01	0.00	<0.001	−0.01	0.00	<0.001
Sex × time	0.09	0.10	0.367	0.07	0.11	0.536	0.01	0.02	0.545	0.02	0.03	0.598	0.04	0.03	0.232	0.01	0.03	0.643
Education × time	0.03	0.02	0.224	−0.06	0.02	0.008	−0.01	0.00	0.092	−0.01	0.01	0.091	−0.01	0.00	0.061	−0.01	0.00	0.138
Activity × time	0.02	0.03	0.595	0.05	0.05	0.337	0.01	0.01	0.173	0.03	0.02	0.145	0.05	0.02	0.025	−0.01	0.02	0.692
Activity change	0.41	0.13	0.002	0.28	0.20	0.164	0.24	0.09	0.006	0.38	0.14	0.005	0.49	0.13	<0.001	0.23	0.12	0.059

	—			LBLS			SLS			VLS								
Fluency										Communication			Computation			Conundrums		
				*b*	SE	*P*	*b*	SE	*P*	*b*	SE	*P*	*b*	SE	*P*	*b*	SE	*P*

Intercept				30.94	0.66	<0.001	36.46	0.46	<0.001	11.68	0.53	<0.001	11.28	0.55	<0.001	11.73	0.53	<0.001
Age				−0.31	0.05	<0.001	−0.34	0.04	<0.001	−0.08	0.02	0.001	−0.08	0.02	0.002	−0.07	0.02	0.006
Sex				3.42	0.93	<0.001	2.88	0.61	<0.001	0.63	0.34	0.065	1.02	0.36	0.005	0.54	0.34	0.115
Education				0.88	0.17	<0.001	1.17	0.11	<0.001	0.47	0.05	<0.001	0.58	0.05	<0.001	0.60	0.05	<0.001
Activity				1.14	0.37	0.002	1.03	0.27	0.001	1.80	0.22	<0.001	0.81	0.22	<0.001	1.68	0.21	<0.001
Age × activity				0.07	0.04	0.121	0.00	0.03	0.900	0.01	0.03	0.709	0.04	0.03	0.112	0.04	0.03	0.094
Education × activity				0.05	0.11	0.651	0.00	0.08	0.995	−0.12	0.06	0.042	−0.04	0.05	0.489	0.03	0.06	0.659
Time				−0.22	0.11	0.052	−0.42	0.04	<0.001	−0.11	0.03	<0.001	−0.13	0.03	<0.001	−0.13	0.03	<0.001
Age × time				−0.02	0.01	0.015	−0.02	0.00	<0.001	−0.01	0.00	0.001	−0.01	0.00	0.001	−0.01	0.00	0.001
Sex × time				−0.17	0.15	0.252	0.11	0.05	0.025	0.07	0.04	0.058	0.08	0.04	0.050	0.07	0.04	0.078
Education × time				0.01	0.03	0.778	−0.01	0.01	0.105	0.00	0.01	0.823	0.00	0.01	0.955	0.00	0.01	0.954
Activity × time				−0.06	0.07	0.350	0.02	0.02	0.324	0.00	0.03	0.868	0.02	0.03	0.448	0.01	0.03	0.793
Activity change				0.49	0.30	0.097	0.50	0.20	0.012	0.76	0.18	<0.001	0.68	0.18	<0.001	0.48	0.16	0.003

	Octo-Twin			LBLS			SLS			VLS								
Memory										Communication			Computation			Conundrums		
	*b*	SE	*P*	*b*	SE	*P*	*b*	SE	*P*	*b*	SE	*P*	*b*	SE	*P*	*b*	SE	*P*

Intercept	8.84	0.29	<0.001	10.73	0.21	<0.001	11.68	0.13	<0.001	17.37	0.41	<0.001	17.06	0.40	<0.001	17.51	0.41	<0.001
Age	−0.18	0.06	0.001	−0.20	0.02	<0.001	−0.18	0.01	<0.001	−0.19	0.02	<0.001	−0.17	0.02	<0.001	−0.18	0.02	<0.001
Sex	0.91	0.99	0.006	1.41	0.30	<0.001	1.40	0.18	<0.001	1.58	0.26	<0.001	2.13	0.27	<0.001	1.50	0.26	<0.001
Education	0.35	0.07	<0.001	0.25	0.05	<0.001	0.26	0.03	<0.001	0.23	0.04	<0.001	0.25	0.04	<0.001	0.30	0.04	<0.001
Activity	0.59	0.10	<0.001	0.17	0.12	0.142	0.41	0.08	<0.001	0.96	0.17	<0.001	1.17	0.16	<0.001	0.85	0.16	<0.001
Age × activity	−0.01	0.04	0.930	0.00	0.01	0.776	0.00	0.01	0.901	0.07	0.02	<0.001	0.06	0.02	0.002	0.06	0.02	0.002
Education × activity	−0.02	0.04	0.663	0.04	0.04	0.247	0.00	0.02	0.948	−0.07	0.04	0.127	−0.02	0.04	0.658	−0.04	0.05	0.345
Time	−0.27	0.07	<0.001	−0.17	0.05	0.001	−0.17	0.01	<0.001	−0.26	0.03	<0.001	−0.26	0.03	<0.001	−0.28	0.03	<0.001
Age × time	0.00	0.02	0.947	0.00	0.00	0.627	−0.01	0.00	<0.001	−0.01	0.00	<0.001	−0.01	0.00	<0.001	−0.01	0.00	<0.001
Sex × time	0.06	0.08	0.465	−0.03	0.07	0.705	0.03	0.02	0.112	−0.01	0.03	0.783	−0.01	0.03	0.736	−0.01	0.03	0.660
Education × time	0.00	0.02	0.932	0.01	0.01	0.691	0.00	0.00	0.549	0.00	0.01	0.349	0.00	0.00	0.349	−0.01	0.00	0.246
Activity × time	0.03	0.02	0.231	0.04	0.03	0.216	0.00	0.01	0.827	0.01	0.02	0.708	0.00	0.02	0.923	0.03	0.02	0.164
Activity change	0.35	0.09	<0.001	0.25	0.12	0.039	0.25	0.07	0.001	0.71	0.13	<0.001	0.78	0.12	<0.001	0.18	0.12	0.126

Semantic knowledge	Octo-Twin			LBLS			SLS			VLS								
										Communication			Computation			Conundrums		
	*b*	SE	*P*	*b*	SE	*P*	*b*	SE	*P*	*b*	SE	*P*	*b*	SE	*P*	*b*	SE	*P*

Intercept	31.60	0.69	<0.001	37.89	0.59	<0.001	24.54	0.23	<0.001	44.23	0.68	<0.001	43.66	0.68	<0.001	44.07	0.69	<0.001
Age	−0.40	0.14	0.004	−0.29	0.05	<0.001	0.01	0.02	0.501	0.09	0.03	0.004	0.11	0.03	<0.001	0.09	0.03	0.004
Sex	−5.17	0.85	<0.001	1.60	0.84	0.056	0.12	0.31	0.688	−0.07	0.44	0.865	0.68	0.46	0.137	−0.18	0.45	0.687
Education	1.60	0.20	<0.001	0.95	0.15	<0.001	0.99	0.06	<0.001	0.58	0.07	<0.001	0.65	0.07	<0.001	0.72	0.07	<0.001
Activity	2.21	0.25	<0.001	0.75	0.34	0.026	0.81	0.14	<0.001	1.97	0.28	<0.001	1.77	0.28	<0.001	1.37	0.27	<0.001
Age × activity	0.03	0.09	0.737	0.05	0.04	0.199	0.02	0.02	0.277	0.05	0.03	0.113	0.06	0.03	0.074	0.07	0.03	0.039
Education × activity	−0.23	0.10	0.014	−0.05	0.10	0.647	−0.05	0.04	0.206	−0.33	0.07	<0.001	−0.28	0.07	<0.001	−0.09	0.08	0.242
Time	−0.92	0.12	<0.001	−0.40	0.09	<0.001	−0.11	0.01	<0.001		0.03	<0.001	−0.14	0.03	<0.001	−0.16	0.03	<0.001
Age × time	−0.05	0.03	0.064	−0.03	0.01	<0.001	−0.01	0.00	<0.001	−0.01	0.00	<0.001	−0.01	0.00	<0.001	−0.01	0.00	<0.001
Sex × time	0.30	0.14	0.036	0.05	0.12	0.678	0.06	0.02	0.001	0.03	0.04	0.379	0.02	0.04	0.533	0.03	0.04	0.439
Education × time	0.04	0.03	0.155	−0.03	0.02	0.185	0.00	0.00	0.447	0.00	0.01	0.510	0.00	0.01	0.725	0.00	0.01	0.453
Activity × time	−0.02	0.04	0.571	0.03	0.05	0.627	0.01	0.01	0.069	0.02	0.02	0.442	−0.01	0.02	0.773	0.01	0.02	0.524
Activity change	0.47	0.17	0.005	0.44	0.20	0.027	0.26	0.07	<0.001	0.49	0.15	0.001	0.53	0.14	<0.001	0.49	0.13	<0.001

Values represent model coefficients and their standard error. Across all studies, time was measured in years since baseline visit, and activity change was entered as a time-varying covariate. All other variables represent baseline measurements alone, in interaction with one another or in interaction with time.
